# Supplementary material for: Analysis of the Origin of Emiratis as Inferred from a Family Study Based on HLA-A, -C, -B, -DRB1, and -DQB1 Genes
Source: Genes (Basel). 2023 May 26;14(6):1159. doi: 10.3390/genes14061159 (PMC10298278; doi:10.3390/genes14061159)
Supplement: Supplementary file 1 [file genes-14-01159-s001.zip › Supplementary Table S2.pdf]

Table S2 Hardy-Weinberg equilibrium for HLA loci

HLA Locus	Sample size	Allele group (N)	Observed heterozygotes	Expected heterozygotes	Chi-square	p-value
A	200	17	178	178.85	0.004	0.9496
B	200	28	180	181.69	0.016	0.8993
C	200	14	174	175.64	0.015	0.9025
DRB1	200	13	169	174.03	0.145	0.7034
DQB1	200	5	149	148.51	0.002	0.9643
