# Supplementary material for: Analysis of the Origin of Emiratis as Inferred from a Family Study Based on HLA-A, -C, -B, -DRB1, and -DQB1 Genes
Source: Genes (Basel). 2023 May 26;14(6):1159. doi: 10.3390/genes14061159 (PMC10298278; doi:10.3390/genes14061159)
Supplement: Supplementary file 1 [file genes-14-01159-s001.zip › Supplementary Table S1.pdf]

Table S1 Reference populations used in the present work

Nº	Populations	Size	References*	Nº	Populations	Size	References*
1	**Emiratis-a**	200	Present study	44	Greece-A	96	[15]
2	Algeria	102	[39]	45	Greece-B	101	[15]
3	Algeria-A	132	[22]	46	Jordan-A	1254	[20]
4	Algeria-B	97	[1]	47	Syria	200	[21]
5	Greece-D	242	[1]	48	Syria-A	225	[30]
6	Tunisia-Gabesia	77	[31]	49	Lebanon	95	[11]
7	Tunisia-Ghannouchia	82	[9]	50	Lebanon-A	1123	[19]
8	Iran-Azeri	100	[50]	51	Lebanon-B	191	[18]
9	Iran-Kurd	100	[50]	52	Lebanon-Armen	368	[4]
10	Tunisia-Berbers Zw	70	[2]	53	Lebanon-KZ	93	[15]
11	Tunisia	376	[33]	54	Lebanon-NS	59	[15]
12	Tunisia-A	80	[32]	55	Lebanese-Y	75	[15]
13	Iran-Khuzestani	50	[42]	56	Palestinians	165	[6]
14	Pakistan-Pathan	100	[51]	57	India-Delhi	112	[53]
15	Pakistan-Sindh	101	[51]	58	Saudi Arabia	105	[5]
16	Tunisia-south	250	[34]	59	Saudi Arabia-A	213	[1]
17	Libya	118	[8]	60	Saudi Arabia-B	158	[23]
18	Libya-Jews	119	[12]	61	Saudi Arabia-C	499	[1]
19	Berbers-Metelsa	99	[35]	62	Saudi Arabia-D	383	[24]
20	Morocco	96	[3]	63	Oman	259	[7] [25]
21	Morocco-A	110	[17]	64	Kuwait	212	[26]
22	Morocco-Agadir	98	[13]	65	Kuwait-A	114	[27]
23	Morocco-Chaouya	98	[36]	66	Bahrain	72	[11]
24	Morocco-Jews	94	[37]	67	Emirates	373	[1]
25	Egypt	101	[15]	68	Iraq- Kurds	209	[28]
26	Egypt-A	121	[14]	69	Yemen-Jews	76	[12]
27	Sudan	200	[1]	70	Yemen-sana'a	50	[29]
28	Greece-Creta	135	[40]	71	Mali-Mossi	42	[15]
29	Spain-Spaniards	176	[16]	72	Senegal-Mandenka	200	[15]
30	Portugal	118	[15]	73	Guinea-Bubi	101	[15]
31	Spain-Murcia	173	[49]	74	Mali-Rimaibe	39	[15]
32	Italy	284	[38]	75	Mali-Fulani	38	[15]
33	Spain-Basques-A	82	[16]	76	Macedonia	172	[47]
34	Spain-Basques-Arratia	83	[46]	77	Turkey	250	[1]
35	Spain-Basques-B	99	[41]	78	Turkey-A	228	[48]
36	France	179	[38]	79	Albania	160	[45]
37	France-Rennes	200	[10]	80	Iran	120	[43]
38	Italy-Sardinia	91	[38]	81	Iran-A	100	[44]
39	Ashkenazi-Jews	132	[37]	82	Iran-Jews	91	[42]
40	Greece-C	98	[15]	83	Iran-Famoori Arabs	84	[42]
41	Bangladesh	141	[1]	84	India-Northeast	188	[52]
42	Thailand	16807	[1]	85	Indonesia	236	[1]
43	Sri Lanka	714	[1]				

*population references are given in Supplementary file

Population references

1. Database of allele frequencies: http://www.allelefrequencies.net, 2019
2. Hajjej A, Sellami MH, Kaabi H, Hajjej G, El-Gaaied A, Boukef K, et al. HLA class I and class II polymorphisms in Tunisian Berbers. Annals of Human Biology. 2011; 38 (2):156-64. https://doi.org/10.3109/03014460.2010.504195 PMID: 20666704
3. Gomez-Casado E, del Moral P, Martinez-Laso J, García-Gómez A, Allende L, Silvera-Redondo C, et al. HLA gene in Arabic-Speaking Moroccans: close relatedness to Berbers and Iberians. Tissue Antigens. 2000; 55(3): 239-49. https://doi.org/10.1034/j.1399-0039.2000.550307.x PMID: 10777099
4. Matevosyan L, Chattopadhyay S, Madelian V, Avagyan S, Nazaretyan M, Hyussian A, Vardapetyan E, Arutunyan R, Jordan F. .HLA-A, HLA-B, and HLA-DRB1 allele distribution in a large Armenian population sample. Tissue Antigens. 2011; 78(1): 21-30. https://doi.org/10.1111/j.1399-0039.2011.01668.x PMID: 21501120
5. Hamdi NM, Al-Hababi FH, Eid AE. HLA class I and class II associations with ESRD in Saudi Arabian population. PLoS One. 2014 Nov 7; 9(11): e111403. https://doi.org/10.1371/journal.pone.0111403 PMD: 25380295
6. Arnaiz-Villena A, Elaiwa N, Silvera C, Rostom A, Moscoso J, Gómez-Casado E, et al. The origin of Palestinians and their genetic relatedness with other Mediterranean populations. Retraction in: Suciu-Foca N, Lewis R. Human Immunology. 2001; 62(9): 889-900. (Accessed on https://commons.wikimedia.org/wiki/File:Palestinians_hla.pdf)
7. Albalushi KR, Sellami MH, Alriyami H, varghese M, Boukef MK, Hmida S. The Investigation of the Evolutionary History of the Omani Population by Analysis of HLA Class I Polymorphism. Anthropologist. 2014; 18(1): 205-210
8. Galgani A, Mancino G, Martínez-Labarga C, Cicconi R, Mattei M, Amicosante M, et al. HLA-A, -B and -DRB1 allele frequencies in Cyrenaica population (Libya) and genetic

relationships with other populations. Hum Immunol. 2013; 74(1): 52-9. https://doi.org/10.1016/j.humimm.2012.10.001 PMID: 23079236

9. Hajjej A, Hmida S, Kaabi H, Dridi A, Jridi A, El Gaaled A, et al. HLA genes in Southern Tunisians (Ghannouch area) and their relationship with other Mediterraneans. European Journal Medical Genetics. 2006; 49(1): 43-56. https://doi.org/10.1016/j.ejmg.2005.01.001. PMID: 16473309

10. Hmida S, Gauthier A, Dridi A, Quillivic F, Genetet B, Boukef K, et al. HLA class II gene polymorphism in Tunisians. Tissue Antigens. 1995; 45(1): 63-8. https://doi.org/10.1111/j.1399-0039.1995.tb02416.x PMID: 7725313

11. Almawi WY, Busson M, Tamim H, Al-Harbi EM, Finan RR, Wakim-Ghorayeb SF, et al. HLA class II profile and distribution of HLA-DRB1 and HLA-DQB1 alleles and haplotypes among Lebanese and Bahraini Arabs. Clinical and Diagnostic Laboratory Immunology. 2004; 11(4): 770-4. https://doi.org/10.1128/CDLI.11.4.770-774.2004 PMID: 15242955

12. Amar A, Kwon OJ, Motro U, Witt CS, Bonne-Tamir B, Gabison R, et al. Molecular analysis of HLA class II polymorphisms among different ethnic groups in Israel. Human Immunology. 1999; 60(8): 723-30. https://doi.org/10.1016/S0198-8859(99)00043-9 PMID: 10439318

13. Izaabel H, Garchon HJ, Caillat-Zucman S, Beaurain G, Akhayat O, Bach JF, et al. HLA class II DNA polymorphism in a Moroccan population from the Souss, Agadir area. Tissue Antigens. 1998; 51(1): 106-10. https://doi.org/10.1111/j.1399-0039.1998.tb02954.x PMID: 9459511

14. Al-Tonbary Y, Abdel-Razek N, Zaghloul H, Metwaly S, El-Deek B, El-Shawaf R. HLA class II polymorphism in Egyptian children with lymphomas. Hematology. 2004; 9(2):139-45. https://doi.org/10.1080/1024533042000205487 PMID: 15203870

15. Clayton J, Lonjou C. Allele and Haplotype frequencies for HLA loci in various ethnic groups. In Charron D, ed. Genetic diversity of HLA. Functional and medical implications. Vol 1. Paris: EDK. 1997; 665-820.

16. Martinez-Laso J, De Juan D, Martinez-Quiles N, Gomez-Casado E, Cuadrado E, Arnaiz-Villena A. The contribution of the HLA-A, -B, -C and -DR, -DQ DNA typing to the study of the origins of Spaniards and Basques. Tissue Antigens. 1995; 45(4): 237-45. https://doi.org/10.1111/j.1399-0039.1995.tb02446.x PMID: 7638859.

17. Brick C, Bennani N, Atouf O, Essakalli M. HLA-A, -B, -DR and -DQ allele and haplotype frequencies in the Moroccan population: a general population study.

18. Samaha H, Rahal EA, Abou-Jaoude M, Younes M, Dacchache J, Hakime N. HLA class II allele frequencies in the Lebanese population. Molecular Immunology. 2003; 39(17-18): 1079-81. https://doi.org/10.1016/S0161-5890(03)00073-7 PMID: 12835080

19. Khansa S, Hoteit R, Shammaa D, Khalek RA, El Halas H, Greige L, et al. HLA class II allele frequencies in the Lebanese population. Gene. 2012; 506(2): 396-9. https://doi.org/10.1016/j.gene.2012.06.063 PMID: 22750800

20. Elbjeirami WM, Abdel-Rahman F, Hussein AA. Probability of finding an HLA-matched donor in immediate and extended families: the Jordanian experience. Biology of Blood and Marrow Transplantation. 2013; 19(2): 221-6. https://doi.org/10.1016/j.bbmt.2012.09.009 PMID: 23025986

21. Mourad J, Monem F. HLA-DRB1 allele association with rheumatoid arthritis susceptibility and severity in Syria. Revista Brasileira De Reumatologia. 2013; 53(1): 47-56. PMID: 23588515

22. Djidjik R, Allam I, Douaoui S, Meddour Y, Cherguelaîne K, Tahiat A, et al. Association study of human leukocyte antigen-DRB1 alleles with rheumatoid arthritis in

Algerian patients. International Journal of Rheumatic Diseases. 2014; https://doi.org/10.1111/1756-185X.12272 PMID: 24447879

23. Hajeer AH, Al Balwi MA, AytülUyar F, Alhaidan Y, Alabdulrahman A, Al Abdulkareem I, Al Jumah M. HLA-A, -B, -C, -DRB1 and -DQB1 allele and haplotype frequencies in Saudis using next generation sequencing technique. Tissue Antigens. 2013; 82(4): 252-8. https://doi.org/10.1111/tan.12200 PMID: 24461004

24. Hajeer AH, Sawidan FA, Bohlega S, Saleh S, Sutton P, Shubaili A, Tahan AA, Al Jumah M. HLA class I and class II polymorphisms in Saudi patients with myasthenia gravis. International Journal of Immunogenetics. 2009; 36(3): 169-72. https://doi.org/10.1111/j.1744-313X.2009.00843.x PMID: 19490212

25. Albalushi KR, Sellami MH, Alriyami H, varghese M, Boukef MK, Hmida S. HLA Class II (DRB1 and DQB1) Polymorphism in Omanis. Journal of Transplantation Technologies and Research 2014; 4: 134. https://doi.org/10.4172/2161-0991.1000134

26. Haider MZ, Shaltout A, Alsaeid K, Qabazard M, Dorman J. Prevalence of human leukocyte antigen DQA1 and DQB1 alleles in Kuwaiti Arab children with type 1 diabetes mellitus. Clinical Genetics. 1999; 56(6): 450-6. https://doi.org/10.1034/j.1399-0004.1999.560608.x PMID: 10665665

27. Haider MZ, Zahid MA, Dalal HN, Razik MA. Human leukocyte antigen (HLA) DRB1 alleles in Kuwaiti Arabs with schizophrenia. American Journal of Medical Genetics. 2000; 96(6): 870-2. https://doi.org/10.1002/1096-8628(20001204)96:6<870::AID-AJMG36>3.0.CO;2-L PMID: 11121200.

28. Arnaiz-Villena A, Palacio-Grüber J, Muñiz E, Campos C, Alonso-Rubio J, Gomez-Casado E, Salih SF, Martin-Villa M, Al-Qadi R. Genetic HLA Study of Kurds in Iraq, Iran and Tbilisi (Caucasus, Georgia): Relatedness and Medical Implications. PLoS One.

2017 Jan 23; 12(1): e0169929. https://doi.org/10.1371/journal.pone.0169929 PMID: 28114347

29. Nassar MY, Al-Shamahy HA, Masood HA. . The Association between Human Leukocyte Antigens and Hypertensive End-Stage Renal Failure among Yemeni Patients. Sultan Qaboos University Medical Journal. 2015; 15(2): e241–249. PMID: 26052458

30. Jazairi B, Khansaa I, Ikhtiar A, Murad H. Frequency of HLA-DRB1 and HLA-DQB1 Alleles and Haplotype Association in Syrian Population. Immunological Investigation. 2016; 45(2): 172-9. https://doi.org/10.3109/08820139.2015.1131293 PMID: 26853713

31. Hajjej A, Hajjej G, Almawi WY, Kaabi H, El-Gaaied A, Hmida S. HLA class I and class II polymorphism in a population from south-eastern Tunisia (Gabes Area). International Journal of Immunogenetics. 2011; 38(3): 191-9. https://doi.org/10.1111/j.1744-313X.2011.01003.x. PMID: 21385325

32. Hajjej A, Kâabi H, Sellami MH, Dridi A, Jeridi A, El borgi W, et al. The contribution of HLA class I and II alleles and haplotypes to the investigation of the evolutionary history of Tunisians. Tissue Antigens. 2006; 68(2):153-62. https://doi.org/10.1111/j.1399-0039.2006.00622.x. PMID: 16866885

33. Hajjej A, Almawi WY, Hattab L, El-Gaaied A, Hmida S. HLA Class I and Class II Alleles and Haplotypes Confirm the Berber Origin of the Present Day Tunisian Population. PLoS One. 2015; 10(8): e0136909. https://doi.org/10.1371/journal.pone.0136909 PMID: 26317228

34. Hajjej A, Almawi WY, Hattab L, El-Gaaied A, Hmida S. The investigation of the origin of Southern Tunisians using HLA genes. Journal of Human Genetics. 2017; 62(3): 419-429. https://doi.org/10.1038/jhg.2016.146 PMID: 27881842

35. Oumhani K, Canossi A, Piancatelli D, Di Rocco M, Del Beato T, Liberatore G, et al. Sequence-Based analysis of the HLA-DRB1 polymorphism in Metalsa Berber and Chaouya Arabic-speaking groups from Morocco. Human Immunology. 2002; 63(2): 129-38. https://doi.org/10.1016/S0198-8859(01)00370-6 PMID: 11821160

36. Canossi A, Piancatelli D, Aureli A, Oumhani K, Ozzella G, Del Beato T, et al. Correlation between genetic HLA class I and II polymorphisms and anthropological aspects in the Chaouya population from Morocco (Arabic speaking). Tissue Antigens. 2010; 76(3): 177-193. https://doi.org/10.1111/j.1399-0039.2010.01498.x PMID: 20492599

37. Roitberg-Tambur A, Witt CS, Friedmann A, Safirman C, Sherman L, Battat S, Nelken D, Brautbar C. Comparative analysis of HLA polymorphism at the serologic and molecular level in Moroccan and Ashkenazi Jews. Tissue Antigens. 1995; 46(2): 104-10 https://doi.org/10.1111/j.1399-0039.1995.tb02485.x PMID: 7482502

38. Imanishi T, Akaza T, Kimura A, Tokunaga K, Gjobori T. Allele and haplotype frequencies for HLA and complement loci in various ethnic groups. In, eds. HLA 1991. VOL 1. Oxford: Oxford University Press.1992; 1065-220.

39. Arnaiz-Villena A, Benmamar D, Alvarez M, Diaz-Campos N, Varela P, Gomez-Casado E, et al. HLA allele and haplotype frequencies in Algerians. Relatedness to Spaniards and Basques. Human Immunology. 1995 ;43(4): 259-68. https://doi.org/10.1016/0198-8859(95)00024-X PMID: 7499173

40. Arnaiz-Villena A, Iliakis P, González-Hevilla M, Longás J, Gómez-Casado E, Sfyridaki K, et al. The origin of Cretan populations as determined by characterization of HLA alleles. Tissue Antigens. 1999; 53(3): 213-26. https://doi.org/10.1034/j.1399-0039.1999.530301.x PMID: 10203014

41. Comas D, Mateu E, Calafell F, Pérez-Lezaun A, Bosch E, Martínez-Arias R, et al. HLA class I and class II DNA typing and the origin of Basques. Tissue Antigens. 1998; 51(1): 30-40. https://doi.org/10.1111/j.1399-0039.1998.tb02944.x PMID: 9459501

42. Farjadian S, Ghaderi A. HLA class II genetic diversity in Arabs and Jews of Iran. Iranian Journal of Immunology. 2007; 4(2): 85-93. https://doi.org/IJIv4i2A3 PMID: 17652848

43. Kollaee A, Ghaffarpor M, Ghlichnia HA, Ghaffari SH, Zamani M. The influence of the HLA-DRB1 and HLA-DQB1 allele heterogeneity on disease risk and severity in Iranian patients with multiple sclerosis. International Journal of Immunogenetics. 2012; 39(5): 414-22. https://doi.org/10.1111/j.1744-313X.2012.01104.x PMID: 22404765

44. Sayad A, Akbari MT, Pajouhi M, Mostafavi F, Zamani M. The influence of the HLA-DRB, HLA-DQB and polymorphic positions of the HLA-DRβ1 and HLA-DQβ1 molecules on risk of Iranian type 1 diabetes mellitus patients. International Journal of Immunogenetics. 2012; 39(5): 429-36. https://doi.org/10.1111/j.1744-313X.2012.01116.x PMID: 22494469

45. Sulcebe G, Sanchez-Mazas A, Tiercy JM, Shyti E, Mone I, Ylli Z, et al. HLA allele and haplotype frequencies in the Albanian population and their relationship with the other European populations. International Journal of Immunogenetics. 2009; 36(6): 337-43. https://doi.org/10.1111/j.1744-313X.2009.00868.x PMID: 19703234

46. Sanchez-Velasco P, Gomez-Casado E, Martinez-Laso J, Moscoso J, Zamora J, Lowy E, et al. HLA alleles in isolated populations from North Spain: origin of the Basques and the ancient Iberians. Tissue Antigens. 2003; 61(5): 384-92. https://doi.org/10.1034/j.1399-0039.2003.00041.x PMID: 12753657

47. Arnaiz-Villena A, Dimitroski K, Pacho A, Moscoso J, Gómez-Casado E, Silvera-Redondo C, et al. HLA genes in Macedonians and the sub-Saharan origin of the Greeks.

Tissue Antigens. 2001; 57(2): 118-27. https://doi.org/10.1034/j.1399-0039.2001.057002118.x PMID: 11260506

48. Arnaiz-Villena A, Karin M, Bendikuze N, Gomez-Casado E, Moscoso J, Silvera C, et al. HLA alleles and haplotypes in the Turkish population: relatedness to Kurds, Armenians and other Mediterraneans. Tissue Antigens. 2001; 57(4): 308-17. https://doi.org/10.1034/j.1399-0039.2001.057004308.x PMID: 11380939

49. Muro M, Marín L, Torío A, Moya-Quiles MR, Minguela A, Rosique-Roman J, et al. HLA polymorphism in the Murcia population (Spain): in the cradle of the archaeologic Iberians. Human Immunology. 2001; 62(9): 910-21. https://doi.org/10.1016/S0198-8859(01)00290-7 PMID: 11543893

50. Farjadian S, Ghaderi A. HLA class II similarities in Iranian Kurds and Azeris. International Journal of Immunogenetics. 2007; 34(6): 457-63. https://doi.org/10.1111/j.1744-313X.2007.00723.x PMID: 18001303

51. Mohyuddin A, Ayub Q, Khaliq S, Mansoor A, Mazhar K, Rehman S, et al. HLA polymorphism in six ethnic groups from Pakistan. Tissue Antigens. 2002; 59(6): 492-501. https://doi.org/10.1034/j.1399-0039.2002.590606.x PMID: 12445319

52. Agrawal S, Srivastava SK, Borkar M, Chaudhuri TK. Genetic affinities of north and northeastern populations of India: inference from HLA-based study. Tissue Antigens. 2008; 72(2): 120-30. https://doi.org/10.1111/j.1399-0039.2008.01083.x PMID: 18721272
