# Supplementary material for: Analysis of the Origin of Emiratis as Inferred from a Family Study Based on HLA-A, -C, -B, -DRB1, and -DQB1 Genes
Source: Genes (Basel). 2023 May 26;14(6):1159. doi: 10.3390/genes14061159 (PMC10298278; doi:10.3390/genes14061159)
Supplement: Supplementary file 1 [file genes-14-01159-s001.zip › Supplementary Table S3.pdf]

Table S3 Complete list of HLA Class I and Class II two-Locus haplotypes in Emiratis

HLA Haplotype	Frequency	SD*	D'	X^2	*P-value*
A-B					
A*02-B*51	0.0750	0.0132	0.21	14.72	0.0001
A*26-B*08	0.0525	0.0112	0.53	85.26	<E-5
A*33-B*58	0.0250	0.0078	0.39	52.30	<E-5
A*03-B*50	0.0250	0.0078	0.25	24.31	<E-5
A*11-B*40	0.0250	0.0078	0.45	40.16	<E-5
A*32-B*35	0.0225	0.0074	0.19	7.22	0.0072
A*24-B*08	0.0200	0.0070	0.16	6.76	0.0093
A*33-B*14	0.0200	0.0070	0.40	42.55	<E-5
A*01-B*37	0.0150	0.0061	0.73	46.42	<E-5
A*03-B*52	0.0150	0.0061	0.38	23.95	<E-5
A*03-B*18	0.0100	0.0050	0.25	9.46	0.0021
A*11-B*41	0.0100	0.0050	0.34	11.10	0.0009
A*01-B*73	0.0075	0.0043	0.38	10.81	0.0010
A*32-B*07	0.0075	0.0043	0.38	12.84	0.0003
A*32-B*18	0.0075	0.0043	0.17	4.70	0.0302
A*26-B*38	0.0075	0.0043	0.56	16.08	6.1 E-5
A*23-B*50	0.0075	0.0043	0.24	6.41	0.0113
A*30-B*42	0.0075	0.0043	0.58	28.94	<E-5
A*24-B*27	0.0050	0.0035	0.46	10.09	0.0015
A*24-B*44	0.0050	0.0035	0.28	5.58	0.0182
A*24-B*49	0.0050	0.0035	0.23	4.32	0.0377
A*66-B*58	0.0050	0.0035	0.65	20.70	<E-5
A*30-B*45	0.0050	0.0035	0.47	15.39	8.7 E-5
A*24-B*39	0.0025	0.0025	0.46	5.02	0.0251
A*01-B*56	0.0025	0.0025	1.00	10.79	0.0010
A*01-B*47	0.0025	0.0025	1.00	10.79	0.0010
A*01-B*81	0.0025	0.0025	1.00	10.79	0.0010
A*74-B*15	0.0025	0.0025	0.23	6.72	0.0095
A*66-B*41	0.0025	0.0025	0.32	11.79	0.0006
A*31-B*42	0.0025	0.0025	0.18	05.03	0.0249
A*23-B*44	0.0025	0.0025	0.15	5.02	0.0251
A*23-B*49	0.0025	0.0025	0.12	4.06	0.0439
A*23-B*57	0.0025	0.0025	0.12	4.06	0.0439
A*11-B*55	0.0025	0.0025	1.00	9.55	0.0020
A*34-B*44	0.0025	0.0025	0.49	32.00	<E-5
A*34-B*15	0.0025	0.0025	0.48	15.26	9.4E-5
A*30-B*53	0.0025	0.0025	0.18	4.95	0.0261

A*30-B*13	0.0025	0.0025	0.29	4.51	0.0337
A*30-B*39	0.0025	0.0025	0.47	7.66	0.0056
A*29-B*53	0.0025	0.0025	0.49	20.84	<E-5
A*29-B*13	0.0025	0.0025	0.50	65.50	<E-5
A*69-B*52	0.0025	0.0025	1.00	27.64	<E-5
B-C					
B*35-C*04	0.1175	0.0161	0.83	238.26	<E-5
B*50-C*06	0.0800	0.0136	0.96	210.04	<E-5
B*51-C*15	0.0700	0.0128	0.43	44.92	<E-5
B*51-C*16	0.0525	0.0112	0.72	62.65	<E-5
B*14-C*08	0.0450	0.0104	1.00	400.00	<E-5
B*58-C*03	0.0450	0.0104	0.77	198.26	<E-5
B*40-C*15	0.0400	0.0098	0.77	83.56	<E-5
B*52-C*12	0.0325	0.0089	0.92	124.57	<E-5
B*51-C*14	0.0300	0.0085	1.00	51.07	<E-5
B*37-C*06	0.0200	0.0070	1.00	51.21	<E-5
B*73-C*15	0.0175	0.0066	1.00	47.68	<E-5
B*49-C*07	0.0175	0.0066	1.00	28.50	<E-5
B*18-C*12	0.0175	0.0066	0.49	33.00	<E-5
B*07-C*07	0.0150	0.0061	0.95	185.92	<E-5
B*41-C*17	0.0150	0.0061	0.59	125.70	<E-5
B*53-C*04	0.0150	0.0061	0.61	18.40	1.8E-5
B*07-C*07	0.0150	0.0061	0.82	19.23	1.2E-5
B*57-C*06	0.0125	0.0056	0.67	19.99	<E-5
B*38-C*12	0.0125	0.0056	1.00	51.20	<E-5
B*42-C*17	0.0125	0.0056	1.00	179.06	<E-5
B*15-C*02	0.0100	0.0050	0.31	39.12	<E-5
B*45-C*16	0.0100	0.0050	1.00	55.82	<E-5
B*15-C*03	0.0075	0.0043	0.20	6.55	0.0105
B*27-C*01	0.0050	0.0035	0.49	77.79	<E-5
B*27-C*02	0.0050	0.0035	0.48	30.67	<E-5
B*44-C*16	0.0050	0.0035	0.29	6.84	0.0089
B*13-C*06	0.0050	0.0035	0.61	7.14	0.0075
B*39-C*12	0.0050	0.0035	1.00	20.32	<E-5
B*56-C*01	0.0025	0.0025	1.00	79.20	<E-5
B*47-C*04	0.0025	0.0025	1.00	5.47	0.0193
B*15-C*05	0.0025	0.0025	0.48	15.26	9.4 E-5
B*81-C*18	0.0025	0.0025	1.00	400.00	<E-5
B*55-C*01	0.0025	0.0025	1.00	79.20	<E-5
B-DRB1					
B*08-DRB1*03	0.0975	0.0148	0.80	117.49	<E-5

B*50-DRB1*07	0.0475	0.0106	0.51	64.97	<E-5
B*52-DRB1*15	0.0300	0.0085	0.84	95.14	<E-5
B*40-DRB1*16	0.0275	0.0082	0.45	20.01	0.00001
B*58-DRB1*03	0.0250	0.0078	0.27	6.36	0.0117
B*14-DRB1*01	0.0150	0.0061	0.30	36.43	<E-5
B*14-DRB1*07	0.0150	0.0061	0.24	7.18	0.0074
B*18-DRB1*16	0.0150	0.0061	0.34	7.42	0.0064
B*37-DRB1*10	0.0125	0.0056	0.61	78.06	<E-5
B*41-DRB1*08	0.0100	0.0050	0.43	66.45	<E-5
B*53-DRB1*04	0.0100	0.0050	0.38	11.70	0.0006
B*49-DRB1*13	0.0100	0.0050	0.55	36.56	<E-5
B*15-DRB1*11	0.0100	0.0050	0.28	13.18	0.0003
B*42-DRB1*03	0.0100	0.0050	0.74	9.76	0.0018
B*51-DRB1*09	0.0075	0.0043	1.00	12.48	4.1E-4
B*73-DRB1*01	0.0075	0.0043	0.40	24.39	<E-5
B*44-DRB1*11	0.0075	0.0043	0.46	17.30	3 E-5
B*57-DRB1*07	0.0075	0.0043	0.34	5.80	0.0160
B*07-DRB1*15	0.0075	0.0043	0.37	8.87	0.0029
B*27-DRB1*08	0.0050	0.0035	0.49	41.89	<E-5
B*45-DRB1*13	0.0050	0.0035	0.47	15.40	0.0001
B*56-DRB1*04	0.0025	0.0025	1.00	8.78	0.0030
B*47-DRB1*13	0.0025	0.0025	1.00	17.22	3 E-5
B*13-DRB1*14	0.0025	0.0025	0.32	13.28	0.0003
B*39-DRB1*13	0.0025	0.0025	0.47	7.66	0.0056
B*42-DRB1*12	0.0025	0.0025	0.19	14.42	15E-5
B*55-DRB1*16	0.0025	0.0025	1.00	4.64	0.0311
DRB1-DQB1					
DRB1*03-DQB1*02	0.2125	0.0205	0.93	204.69	<E-5
DRB1*16-DQB1*05	0.1725	0.0189	0.96	187.82	<E-5
DRB1*07-DQB1*02	0.1100	0.0157	0.79	76.03	<E-5
DRB1*04-DQB1*03	0.0875	0.0141	0.82	119.94	<E-5
DRB1*15-DQB1*06	0.0850	0.0140	0.85	192.21	<E-5
DRB1*11-DQB1*03	0.0650	0.0123	0.91	98.28	<E-5
DRB1*13-DQB1*06	0.0475	0.0106	0.84	101.25	<E-5
DRB1*01-DQB1*05	0.0450	0.0104	1.00	44.51	<E-5
DRB1*10-DQB1*05	0.0375	0.0095	1.00	36.80	<E-5
DRB1*14-DQB1*05	0.0225	0.0074	1.00	21.74	<E-5
DRB1*08-DQB1*04	0.0125	0.0056	0.54	80.11	<E-5
DRB1*04-DQB1*04	0.0100	0.0050	0.22	6.15	0.0131
DRB1*12-DQB1*03	0.0100	0.0050	0.75	11.19	0.0008
DRB1*09-DQB1*03	0.0075	0.0043	1.00	11.90	0.0006
