# Supplementary material for: Analysis of the Origin of Emiratis as Inferred from a Family Study Based on HLA-A, -C, -B, -DRB1, and -DQB1 Genes
Source: Genes (Basel). 2023 May 26;14(6):1159. doi: 10.3390/genes14061159 (PMC10298278; doi:10.3390/genes14061159)
Supplement: Supplementary file 1 [file genes-14-01159-s001.zip › Supplementary Table S4.pdf]

Table S4 Ewens-Watterson homozygosity test of neutrality

HLA Locus	Observed F	Expected F	Normalized deviate of F (Fnd)	p-value
A	0.1058	0.2176	-1.3629	0.0064
B	0.0916	0.1287	-0.8502	0.1536
C	0.1218	0.2620	-1.3993	0.0030
DRB1	0.1299	0.2806	-1.4191	0.0051
DQB1	0.2574	0.5696	-1.6801	0.0064
